# Analytical optimization of open hole effects on the tensile properties of SS400 sheet specimens using an integrated FFD-CRITIC-DFA method

**DOI:** 10.1016/j.heliyon.2023.e23920

**Published:** 2023-12-20

**Authors:** Teerapun Saeheaw

**Affiliations:** Department of Teacher Training in Mechanical Engineering, King Mongkut's University of Technology North Bangkok, Bangkok, Thailand

**Keywords:** Parameter optimization, Open holes, Tensile properties, SS400 steel, Yield strength, Ultimate tensile strength, Ultimate elongation

## Abstract

Structural components are generally composed of material discontinuities, including open holes, which are considered stress concentrators in engineering components. In view of this, assessing the influence of open holes on the tensile properties is crucial to determine the sensitivity and tensile strength of a particular material. Nevertheless, investigation of the impact of open holes on the tensile properties of SS400 steel sheets is very limited and yet to be explored. Therefore, this study was performed to optimize the effects of open holes on the tensile properties of SS400 sheet specimens based on a Full Factorial Design (FFD) experiment. Four input parameters that represent various hole configurations, which include the hole diameter, location of the hole, number of holes, and hole shape, were considered in this study to develop the experimental-based prediction models to optimize the output performance, namely yield strength, ultimate tensile strength, and ultimate elongation, commonly denoted as YS, UTS, and UE respectively. A total of 10 additional experimental trials were then utilized to verify the constructed models. In addition, the weight fractions for YS, UTS, and UE were identified using the Criteria Importance Through Inter-Criteria Correlation (CRITIC) method. Subsequently, the Desirability Function Analysis (DFA) is utilized to pinpoint the optimal parameter conditions for maximizing the tensile properties. Based on the results, all four parameters showed significant effects on the response variables, except the number of holes for UTS and hole location for UE. The diameter also recorded the highest contribution toward UTS and UE, followed by the hole shape. Regarding YS, hole diameter takes precedence, with the number of holes as the second most influential factor. Furthermore, the average absolute percent deviation for the prediction responses of 10 experimental cases were 1.06 %, 0.90 %, and 0.85 % for YS, UTS, and UE, respectively, confirming the validity of the constructed models. Meanwhile, the CRITIC method estimated the weight fractions for YS, UTS and UE from the experimental data, which were 0.3825, 0.2559, and 0.3616, respectively. The DFA-derived composite desirability, rated at 0.9820, suggests optimal conditions: a 1 mm hole diameter, centered hole location, three holes, and a hexagonal shape. The minimal deviations between predicted and experimental values affirm the robustness of the models. Overall, this investigation yields important insights for optimizing open holes and elevating the tensile performance of SS400 sheet specimens.

## 1 Introduction

Incorporating open holes into the structural design of practical applications, such as access points and connections, is crucial. This integration leads to stress concentration around the hole boundary, resulting in a reduction of the tensile load capacity of the component. The assessment of hole-induced stress concentration in flat specimens with a central hole is typically measured through the notched tensile strength [1]. However, in most construction designs, the connection between structural components involves more than a single bolt. This results in a progressively intricate stress field, as bypass stresses can be transferred to neighboring bolts [2,3]. Additionally, the use of multiple staggered holes, often arranged in rivet patterns, reduces the tensile strength of a tension member compared to a single row of holes. It's noteworthy that failure does not typically occur at both rows of holes. However, the sections parallel to the holes in the extra row struggle to sustain the full load share, leading to an uneven stress dispersion across the entire section [4,5].

The presence of open holes induces stress concentrations primarily due to geometric factors rather than material properties [5]. The size and spacing of the holes influence elastic stress concentration, while plastic stress concentrations are alleviated through stress redistribution based on the material's plastic properties. Ductile materials exhibit greater stress redistribution and allow stress buildup in other sections before failure, contributing to a more gradual failure process. In contrast, brittle materials sustain high-stress concentrations just before breaking, resulting in a significant reduction in ultimate strength [5]. Ductile materials find extensive application in structural scenarios owing to their diminished notch sensitivity and the incorporation of apertures. Prior investigations have demonstrated a significant reduction in the strength of materials prone to notching when exposed to notches, cutouts, holes, or other structural irregularities [6].

Investigating the influence of open-hole stress concentration, Mallick [1] studied the tensile strength of an SMC-R50 composite molding sheet, which had a nominal thickness of 2.7 mm. Considering the location of the centric and eccentric holes with a diameter range of 3.3–12.7 mm, the findings indicated that an increased hole eccentricity led to a more significant reduction in the notched tensile strength of the R50 material. In a separate investigation, Komurlu and Demir [7] explored the use of the drilled core specimen testing method to assess the tensile strength of rock materials. The study noted that the hole drilling location was also a crucial parameter that significantly impacted the stress distribution. In their study, Eugene and colleagues [8] explored the tensile strength of a composite laminate featuring multiple holes. Their findings revealed that the notched strength of the double-column plate surpassed that of its single-column plate counterpart. This outcome suggests that the additional hole in the adjacent column, aligned across the loading direction of the plate, plays a crucial role in maximizing stress reduction through stress relief mechanisms. Furthermore, the study inferred that the staggered configurations exhibited a lower notched strength compared to non-staggered configurations.

Based on the available evidence, ductile materials, such as the SS400 steel, could always experience a certain stress concentration level near the discontinuity. As a result, the decreased elongation not only diminishes impact strength but also impacts the performance of interconnected components [9]. Failure initiation at the perforation edge tends to compromise strength due to stress concentration [10]. Evaluating the influence of open holes on tensile properties is crucial for assessing material sensitivity [11].

In this study, the responses chosen for optimization are the yield strength, ultimate tensile strength, and ultimate elongation, commonly denoted as YS, UTS, and UE respectively, of SS400 sheet specimens. The decision to focus on maximizing these specific mechanical properties is rooted in several key considerations, which contribute to the comprehensive understanding of material behavior and performance.

Firstly, YS represents the stress at which the material undergoes plastic deformation. Maximizing YS is essential because it ensures that the material can withstand higher levels of stress without permanent deformation. In structural engineering and many industrial applications, a high YS is highly desirable as it guarantees the safety and integrity of components under load.

Secondly, UTS denotes the peak stress that a material can sustain before reaching the point of failure. Maximizing UTS is crucial because it signifies the material's ability to resist external forces and prevents premature rupture or failure. This is particularly important in situations where materials need to withstand extreme loads or conditions.

Thirdly, UE measures the material's ability to stretch or deform before breaking. While maximizing UE may not always be the primary objective, it is essential in applications where ductility and deformation capacity are critical. For instance, in scenarios involving forming or shaping processes, materials with high elongation can be more easily molded without fracturing.

The decision to maximize these responses is guided by the need to ensure the structural and mechanical integrity of SS400 sheet specimens, especially when subjected to various hole configurations. By optimizing YS, UTS, and UE, we strive to enhance the overall performance and reliability of the material, making it well-suited for a broader range of applications.

Furthermore, focusing on the maximization of these properties aligns with the engineering principles of safety, reliability, and efficiency. It allows for the development of SS400 sheet specimens that can withstand stress concentrations caused by open holes, ensuring their suitability for demanding tasks in the construction and machinery industries.

Parametric optimization of open-hole configurations is vital to maximizing the tensile properties of a material, which includes the YS, UTS, and UE. Therefore, experimental optimization was performed to assess the impact of individual and combined parameters. This process involved developing a mathematical model through the application of design of experiments (DOE), a statistical technique that correlates input parameters with the desired objective function. The parametric optimization is then executed via the developed mathematical model. Since multi-objective optimizations require the determination of weight fraction for individual objective functions, the Criteria Importance Through Inter-Criteria Correlation method, CRITIC for short, is employed to ascertain weight fractions for multi-objective functions. Its enhanced flexibility in scientific weight assignment, based on the variability in parametric values, makes it a preferred choice [12].

Desirability Function Analysis (DFA) is a statistical tool utilized across a range of domains [13]. Its primary role lies in optimizing parameters by simultaneously assessing multiple factors. DFA excels at identifying combinations of factors that maximize desired outcomes. In experimental design, DFA significantly improves the process by pinpointing optimal factor settings and evaluating the overall quality of parameter combinations, thereby yielding more informative results. This is particularly valuable in fields such as engineering, and manufacturing [14,15]. Moreover, DFA is an invaluable tool in material science, enabling the efficient optimization of factors that influence material properties [16].

Despite the abundance of theoretical and experimental reports on the effects of open holes in composite materials, limited studies have delved into the impact of open holes on metal sheets. Furthermore, none have elucidated the input–output relationships of open holes and the tensile properties (YS, UTS, and UE). Recognizing this research gap, our study is dedicated to optimizing the effects of open holes on the tensile properties of SS400 sheet specimens. The primary contributions of our investigation are outlined below.

• The exploration of FFD-based regression modeling to understand the comprehensive quadratic effects (linear, square, and interaction) of factors related to hole configurations (such as hole diameter, location, number, and shape) on the tensile properties (YS, UTS, and UE). This aspect has not been previously investigated.
• The development of empirical response equations, based on experimental data, to predict the impact of hole configurations on the tensile properties. This area of research has not been explored before.
• Conducting real-world tests to confirm the practical utility of the developed models in an industrial context.
• Investigating the relationship between hole configurations and their influence on the tensile properties.
• Determining the weight fractions assigned to each objective function.
• Performing multi-objective optimization (considering YS, UTS, and UE) using DFA for assessing the impact of hole configurations on the tensile properties.

In alignment with our study's contributions, our research aims to comprehensively investigate the effects of open holes on the tensile properties of SS400 sheet specimens. To provide a clearer perspective on our research goals, we have refined the presentation of our research objectives and the underlying rationale. By addressing the identified research gap, our study offers novel insights into the intricate relationships between hole configurations and their impacts on YS, UTS, and UE.

**Fig. 1:**
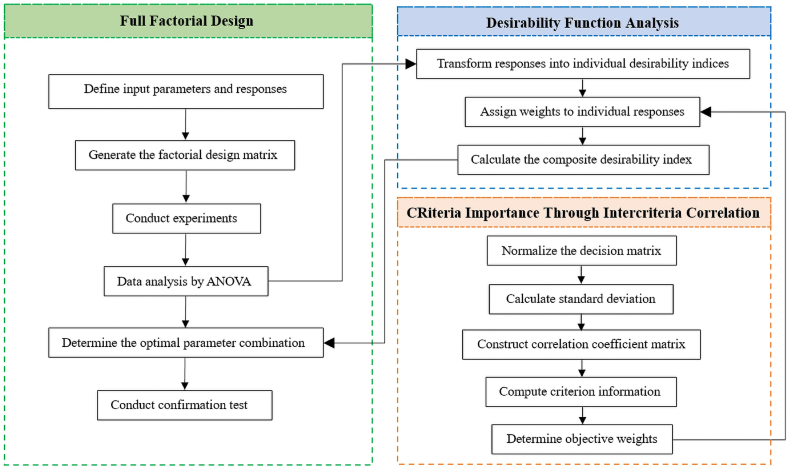
Flow chart of an integrated FFD-CRITIC-DFA method.

Our methodology employs an integrated FFD-CRITIC-DFA method, a systematic and structured framework comprising several phases, as illustrated in Fig. 1. These phases include parameter definition, experimentation, data analysis utilizing Analysis of Variance (ANOVA), CRITIC assessment, and DFA optimization. Each phase is crucial in achieving research goals. It allows for a comprehensive exploration of the problem space and facilitates informed decision-making to find the optimal parameter combination for SS400 sheet specimens.

In light of the valuable insights derived from our study, it's imperative to recognize specific limitations. Firstly, while our findings are relevant to a range of hole configurations, their direct applicability to bolted or riveted joint scenarios may be limited. Secondly, as with any experimental study, variations may exist in real-world conditions that our controlled experiments do not fully capture.

2 3 4 5

### 1.1 Literature review

This subsection is divided into two subsections, providing a condensed overview of MCDM techniques and then shifts its focus to the practical applications of optimization algorithms tailored to our specific problem.

### 1.2 MCDM techniques

The existing body of literature classifies weighting methods into two primary categories: subjective and objective approaches [17,18]. Subjective methods rely on information initially provided by decision-makers, often drawing from their expertise or experience [19]. Examples of common subjective weighting methods encompass pairwise-comparison-based techniques [20,21], stepwise weight assessment ratio analysis (SWARA) [22], simple multi-attribute rating technique using similarity (SIMOS) [23], Swinburne weighted indexing method for gauging (SWING) [24], and decision-making trial and evaluation laboratory (DEMATEL) [25], among others. While subjective methods integrate information from experienced decision-makers, they may introduce bias if decision-makers have personal preferences or incomplete knowledge of the decision problem [26,27]. Moreover, the complexity of collecting and presenting such information can escalate, especially when multiple criteria are implicated in the MCDM problem.

In contrast, objective methods operate without requiring any initial input or judgment from decision-makers [28]. They analyze the inherent structure of available data in the decision matrix to derive weights [[29], [30], [31]]. Renowned for their capacity to eliminate potential bias associated with subjective evaluations, these methods enhance objectivity [32]. Notable examples of objective methods encompass the entropy method [33,34], CRITIC [35], multiple attribute decision-making by ratio and equilibrium (MEREC) [36], and logarithmic percentage change-driven objective weighting (LOPCOW) [37]. Within the spectrum of objective methods, the entropy method and CRITIC emerge as widely applied techniques for weighting criteria. CRITIC holds a distinct advantage by considering both contrast intensity and conflicting relationships between decision criteria [38,39], a feature absent in the Shannon entropy method, which solely addresses contrast intensity [40]. Further elaboration on these aspects is provided below.

The variability of individual criteria's local scores is a crucial aspect of decision-making contrast. In the CRITIC methodology, this contrast intensity is determined by evaluating the standard deviation (SD) for each criterion [41]. This methodology ensures that criteria displaying higher contrast intensity or SD are assigned greater weights. This is because criteria with more significant variance in scores between alternatives contribute more meaningful information [42]. As a result, decision-makers should give increased attention or assign higher weights to such criteria in comparison to those with uniform scores.

In MCDM problems, the presence of conflicting criteria is a common challenge, as it becomes impractical for any alternative to fully satisfy all predetermined criteria [43]. The existence of conflicting relationships signifies the intricate nature of the associations between decision criteria. The CRITIC method addresses these conflicts by incorporating the Pearson correlation coefficient [44], which spans from −1 to 1. A coefficient of zero signifies independence between the two criteria, allowing for a nuanced examination of their relationship, while a negative coefficient indicates the opposite directions followed by the criteria. As the coefficient tends towards −1, the divergence between the criteria intensifies. In contrast, a positive coefficient indicates a congruent directionality between the criteria, indicating redundancy in the information provided by them [45]. The CRITIC method, guided by particular formulas, attributes more weight to criteria characterized by heightened conflict or diminished redundancy.

Overall, the CRITIC method allocates greater weight to criteria characterized by heightened contrast intensity and more pronounced conflicts with other criteria [46]. This aspect has led to the widespread use of CRITIC in numerous real-world applications.

### 1.3 Optimization algorithms

Selecting the most appropriate optimization technique for a specific application remains a challenge, as there's no universal standard. Each algorithm has its unique strengths and limitations, tailored to specific problem types. An effective optimization algorithm converges to a global minimum with minimal iterations. This opens up an opportunity to apply various optimization techniques and compare their performance to find the best parameter conditions. Scholars have utilized various optimization techniques, including genetic algorithms (GA), particle swarm optimization (PSO), harmony search (HS), artificial bee colony (ABC), ant colony optimization (ACO), biogeography-based optimization (BBO), and teaching–learning-based optimization (TLBO), to tackle and enhance solutions for real-world challenges [47,48]. These choices are based on the unique qualities of each algorithm and their suitability for different problem types. Researchers intentionally select these algorithms due to their ability to balance exploration and exploitation, navigate complex solution spaces, and draw inspiration from natural processes. This strategic selection aligns algorithms with each problem's characteristics, achieving the desired trade-off between exploration and exploitation. Furthermore, these techniques offer cost and time efficiencies and are highly reproducible, solidifying their value in optimization studies.

For our specific problem involving quadratic equations and its relative simplicity, using metaheuristic algorithms and their variants may unnecessarily complicate matters. Multi-objective algorithms like NSGA-II, NSGA-III, MOPSO, MOHS, and MOABC are designed for more complex scenarios [49] and might not be suitable for our straightforward problem. In this context, analytical methods can provide precise solutions. DFA aligns well with our research objectives, particularly when optimizing relatively simple problems characterized by quadratic equations. Therefore, employing streamlined algorithms like DFA is more suitable for the scale and nature of this problem, ensuring computational efficiency and a straightforward solution process. DFA proves to be a valuable tool for identifying optimal parameter settings in multi-objective optimization across various industries. It is widely favored for its simplicity and adaptability [50,51], extending its applicability across diverse fields of science and research [[13], [14], [15]]. It is a suitable choice whenever simultaneous optimization of multiple responses is required [13]. DFA's primary objective is to identify an input variable set that aligns all responses with target values as closely as possible. This methodology is versatile, addressing both continuous and discrete design space challenges [16] and effectively tackling multi-dimensional optimization problems in specific cases.

In summary, for a problem characterized by a simple quadratic model and a small scale, the mathematical structure is clear, and the problem is well-suited for an analytical approach like DFA. This method leverages the exact mathematical model to find the optimal solution, ensuring precision. On the other hand, metaheuristics, designed for more complex and poorly-structured problems, may find good approximations but are less likely to reach the exact solution, especially when the problem's characteristics do not fully challenge their search and optimization capabilities. In cases where an analytical solution is feasible, as in a simple quadratic model, selecting the appropriate optimization method becomes even more critical, and DFA is often the preferred choice for obtaining precise results.

The study unfolds as follows: Section 2 outlines the materials and methodologies applied in this study, while Section 3 provides a thorough analysis and discussion of the results obtained from each method. Lastly, Section 4 presents the conclusion.

## 2 Materials and methods

The materials and methods section details the research approach and techniques employed to optimize the effects of open holes on the tensile properties of SS400 sheet specimens using an integrated FFD-CRITIC-DFA method.

The flowchart depicted in Fig. 1, referred to as an integrated FFD-CRITIC-DFA method, serves as a systematic framework meticulously designed to undertake a comprehensive analysis and optimization of the impact of open holes on the tensile properties of SS400 sheet specimens. This methodical approach ensures a thorough exploration of the problem space and facilitates well-informed decision-making throughout the research process. To initiate this approach, the first crucial step involves defining the input parameters and responses. These parameters and responses serve as the foundation for the subsequent phases of the methodology, allowing for a structured and methodical exploration of the problem. Following the initial setup, the generation of a FFD matrix takes place. This matrix serves as a systematic arrangement of experimental conditions, providing a comprehensive coverage of potential configurations related to hole diameter, location, number, and shape. It forms the basis for conducting experiments in the subsequent phase, ensuring that all feasible combinations of these parameters are systematically examined. Subsequently, data collection ensues through the systematic variation of input parameters as defined in the factorial design matrix. This empirical data serves as the bedrock for subsequent analyses and optimization steps. Moving forward, data analysis is conducted using ANOVA. ANOVA serves as a robust statistical tool to rigorously assess the influence of input parameters on response variables, guaranteeing the reliability of the developed models and facilitating meaningful insights from the experimental data. Following the data analysis, the CRITIC methodology is employed. Initially, the decision matrix is normalized to establish a common scale for all criteria, simplifying subsequent calculations. The standard deviation is then calculated to quantify the variability of each response variable, providing valuable insights into data dispersion. Subsequently, a correlation coefficient matrix is constructed, systematically assessing the relationships between response variables. This step enhances the understanding of how these variables interact and influence each other, contributing to more informed decision-making. The determination of criterion information follows, enabling the assignment of importance to each objective or response variable. This foundational step forms the basis for calculating objective weights, reflecting the relative significance of each objective in guiding the optimization process. Transitioning to the DFA phase, responses are transformed into individual desirability indices. These indices provide a clear assessment of how well each response variable aligns with its respective target or ideal value. CRITIC weights, derived from earlier analysis, are thoughtfully assigned to responses, considering the relative importance of each response variable in the optimization process. Finally, the composite desirability is computed, amalgamating individual desirability indices while factoring in their respective CRITIC weights. This composite desirability score acts as a comprehensive measure guiding the optimization process towards the identification of the optimal parameter combination.

In summary, an integrated FFD-CRITIC-DFA method represents a meticulously designed framework that systematically guides the comprehensive analysis and optimization of the effects of open holes on the tensile properties of SS400 sheet specimens. This method ensures a methodical exploration of the problem space, from defining input parameters to the final identification of the optimal parameter combination. In short, it provides a structured and data-driven approach that facilitates well-informed decision-making throughout the research process, ultimately leading to valuable insights for optimizing the behavior of SS400 sheet specimens with open holes.

### 2.1 Materials and experimental setup

This study utilized the low-carbon SS400 steel sheet as it is considered one of the most demanding and dominantly used hot-rolled structural steel in the construction and machinery industries. Table 1, Table 2 present the chemical composition and mechanical properties of the SS400 specimen, respectively.

**Table 1:** Chemical composition of the SS400 steel specimen (wt%).

C	Mn	Si	P	S	Al	Cu	B	Cr	Mo	V	Ni	CrM	CMN
0.044	0.161	0.004	0.008	0.006	0.050	0.007	0.0000	0.016	0.002	0.002	0.023	0.018	0.048

**Table 2:** Mechanical properties of the SS400 steel specimen.

Yield Strength (MPa)	Tensile Strength (MPa)	Elongation (%)
289	363	37

**Fig. 2:**
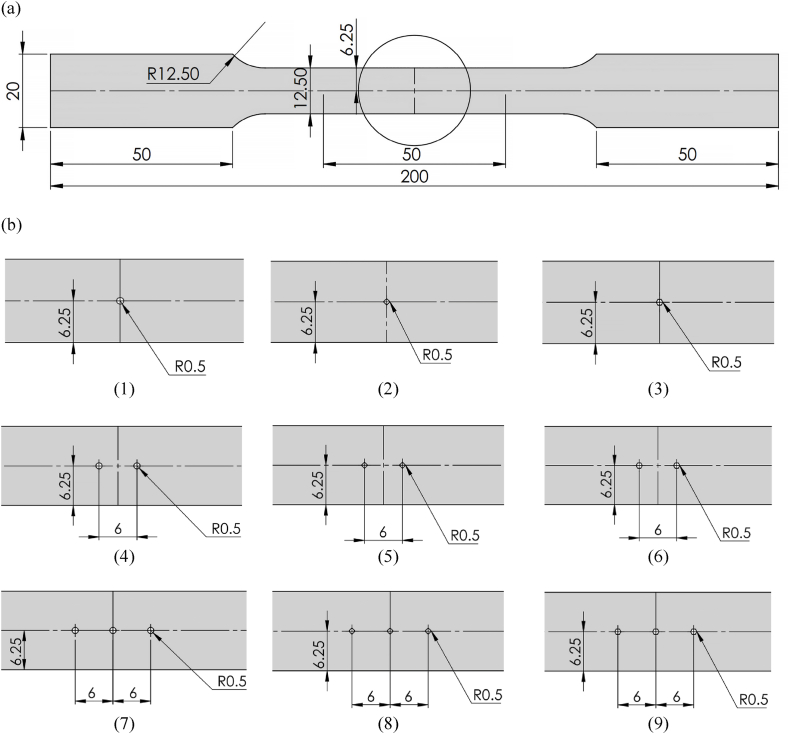

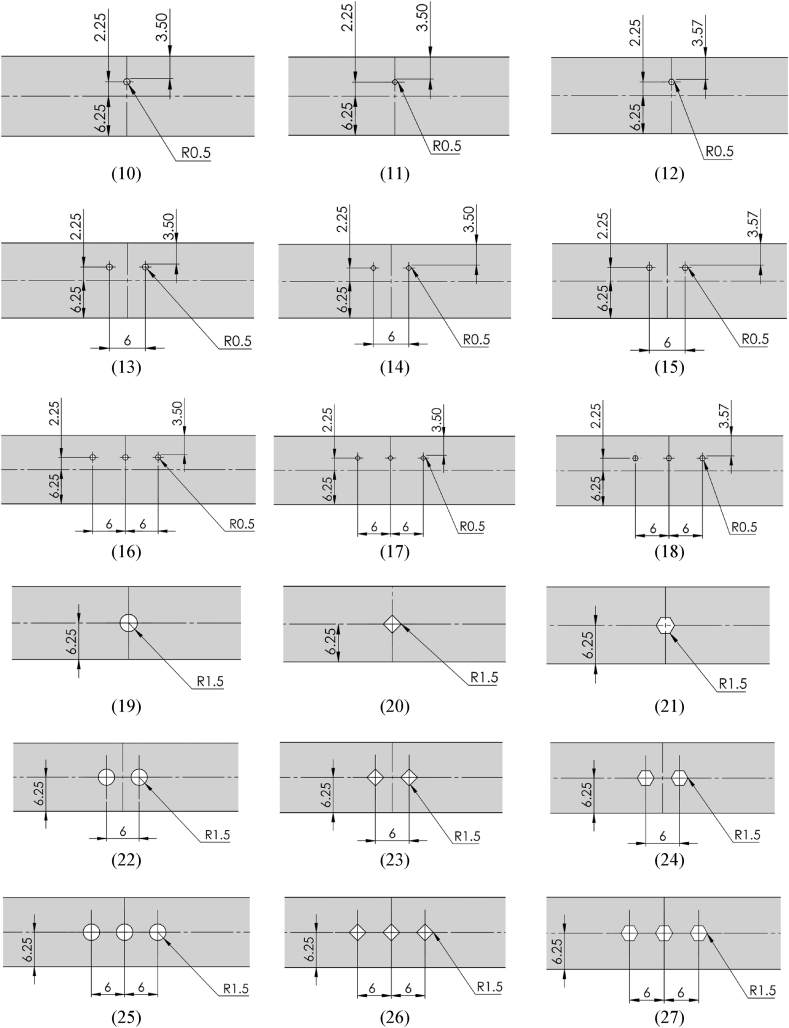

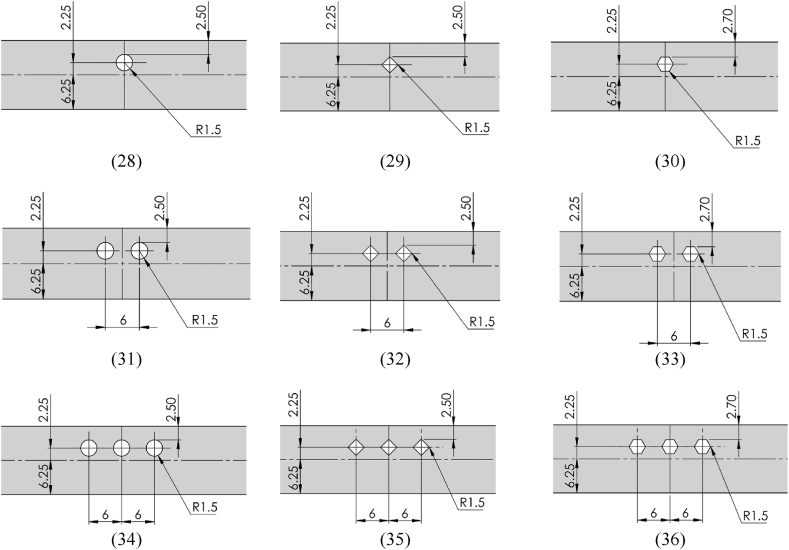
Schematic dimensions and geometry of the tensile specimens (measured in mm). (a) Smooth tensile specimen. (b) Notched tensile specimens with 36 subpanels. Fig. 2: Schematic dimensions and geometry of the tensile specimens (measured in mm). (a) Smooth tensile specimen. (b) Notched tensile specimens with 36 subpanels. (cont.) Fig. 2: Schematic dimensions and geometry of the tensile specimens (measured in mm). (a) Smooth tensile specimen. (b) Notched tensile specimens with 36 subpanels. (cont.)

A laser-cutting machine was used to cut the sheet specimen into smaller sizes with a dimension of 12.5-mm width, 50-mm gauge length, and 1-mm thick, following the American Society for Testing and Materials (ASTM) E8 standard. Prior to the tensile test, the gauge length was marked on the flat smooth specimens, as illustrated in Fig. 2a, and on the specimens with open holes, as depicted in Fig. 2b. In addition, holes were subdrilled and subsequently reamed to ensure the consistency of each hole. A micrometer caliper was used to accurately measure the width and thickness of the specimen prior to the test, while the hole diameters were determined using plug gauges.

### 2.2 Experimental design matrix

Based on a detailed literature review [[1], [2], [3], [4], [5], [6], [7], [8], [9], [10], [11]], four input parameters were selected as independent variables to study the effect of open holes on the tensile properties of the SS400 steel specimen, comprising hole diameter, location, number, and shape (designated as parameters A, B, C, and D, respectively). The hole locations consisted of (1) centric and (2) eccentric locations, where the latter location represents various practical conditions, especially with structures containing holes that may be positioned off-center relative to the loading direction [1]. Meanwhile, the hole shape included (1) circle, (2) square, and (3) hexagon, which were selected based on their relevance to the drilling assembly of holes during the joining operations. The diameter size of 1 and 3 mm were chosen to ensure the holes were within the practical range of the applied machine structure. In addition, a square and a regular hexagonal shape were inscribed in a circle with diameters of 1 and 3 mm, respectively (Table 3). The area of the circle $\pi r^{2}$ has a diameter equal to the length of the diagonal of the square. Thus, the area of the square becomes $2r^{2}$. The radius of the circle is also equal to the side of the hexagon, which consists of six equilateral triangles. Hence, the area of the hexagon is $6\times \sqrt{3}/4\times r^{2}$. Based on these descriptions, the area ratio of the circle, square, and hexagon is $2\pi :4:3\sqrt{3}$.

**Table 3:** Input parameters and levels of hole configurations.

Parameter	Notation	Levels (low, medium, and high)
Hole diameter, in mm	*A*	1 and 3
Hole location	*B*	1 and 2
Number of holes	*C*	1, 2 and 3
Hole shape	*D*	1, 2 and 3

The tensile properties of the SS400 steel specimen were assessed by conducting a parametric study of the open holes according to the FFD-based experiment to obtain the most precise output using the four factors and levels, as presented in Table 3. Additionally, all the specimens were designed according to the sheet metal design guidelines, as illustrated in Fig. 2b comprising 36 subpanels. The numbering of specimens in each subpanel aligns with the experiment number in the respective row of the design matrix outlined in Table 4. This meticulous design ensures consistency and traceability in the experimental setup, facilitating a comprehensive analysis of the obtained results. It was recommended that the hole diameter should be the same or more than the thickness of the sheet. Should the holes be placed closer to each other or the edge of sheets, it was recommended that the minimum distance should be at least 2 times and 2.5 times the material thickness between each hole and between the hole and the edge of the sheet, respectively.

**Table 4:** Design matrix with response values based on the FFD experiments.

Exp. no	Input parameters				Responses		
	A (mm)	B	C	D	YS (MPa)	UTS (MPa)	UE (%)
1	1	1	1	1	285	301	24.36
2	1	1	1	2	282	330	26.03
3	1	1	1	3	288	340	28.62
4	1	1	2	1	274	326	24.43
5	1	1	2	2	271	328	26.90
6	1	1	2	3	276	328	28.64
7	1	1	3	1	281	330	26.07
8	1	1	3	2	287	334	27.75
9	1	1	3	3	283	340	27.77
10	1	2	1	1	281	330	24.36
11	1	2	1	2	282	336	26.92
12	1	2	1	3	275	326	26.94
13	1	2	2	1	269	325	25.24
14	1	2	2	2	266	327	26.86
15	1	2	2	3	274	331	27.75
16	1	2	3	1	274	329	25.24
17	1	2	3	2	286	337	27.75
18	1	2	3	3	280	341	28.62
19	3	1	1	1	270	287	7.27
20	3	1	1	2	275	291	5.55
21	3	1	1	3	284	313	13.25
22	3	1	2	1	258	290	8.14
23	3	1	2	2	260	290	8.13
24	3	1	2	3	262	298	14.98
25	3	1	3	1	245	286	10.71
26	3	1	3	2	254	285	8.99
27	3	1	3	3	257	293	14.12
28	3	2	1	1	268	283	6.44
29	3	2	1	2	269	284	6.44
30	3	2	1	3	282	289	12.40
31	3	2	2	1	254	282	8.99
32	3	2	2	2	253	287	9.76
33	3	2	2	3	261	294	14.10
34	3	2	3	1	240	278	11.53
35	3	2	3	2	251	282	9.84
36	3	2	3	3	253	295	17.52

A Cometech QC-506M1 Universal Testing Machine (UTM) was employed to evaluate the three response variables, specifically an ordinary 0.2-offset YS, UTS, and UE, via the uniaxial tensile test with a maximum capacity of 20 kN and 50 mm/min constant crosshead speed. Each parameter configuration was analyzed using two replicates to obtain the mean values. Since this study was focused on the plastic behavior of the material, the elastic modulus of the test specimens that represents the elastic behavior was beyond the scope of this study and was therefore, not included. Table 4 lists the design matrix comprising the entire set of 2 × 2 × 3 × 3 = 36 conditions for the FFD experiments.

### 2.3 Regression modeling of YS, UTS and UE

This section elucidates the methodology employed to analyze the effects of critical factors on YS, UTS, and UE in SS400 sheet specimens. The chosen approach is firmly grounded in the principles of FFD, with the primary objective of comprehensively understanding the intricate relationships between these critical factors and the tensile properties under investigation.

The FFD-based regression modeling approach systematically varies the levels of critical factors, including hole diameter, location, number, and shape. This systematic variation is essential to enable a thorough analysis of their effects on the response variables (YS, UTS, and UE). FFD, by design, permits the exhaustive exploration of the entire parameter space by testing all possible combinations of factor levels. This comprehensive approach also allows for the capture of quadratic effects, which are pivotal for understanding nonlinear relationships between factors and responses.

To implement FFD-based regression modeling, a series of meticulously designed experiments is conducted, with each factor systematically manipulated across multiple levels. These experimental runs generate a dataset comprising corresponding responses, forming the foundation for constructing regression models. These models establish mathematical relationships between the factors and responses, encompassing linear, quadratic, and interaction terms. The process of fitting the regression equations to the experimental data yields model coefficients, which provide valuable insights into the strength and direction of each factor's influence on the responses.

In this study, we investigated four critical input factors: hole diameter, location, number, and shape, with YS, UTS, and UE as the response variables. By including quadratic terms in the regression models, we capture curvature and nonlinearity in the relationships, enabling a deeper understanding of how changes in factors interact and contribute to variations in the responses.

(1) $$Y_{i}=\beta_{0}+\sum_{i=1}^{k}\beta_{i}x_{i}+\sum_{i=1}^{k}\beta_{ii}x_{i}^{2}+\sum_{i=1}^{k}\sum_{j>i}\beta_{ij}x_{i}x_{j}+\varepsilon_{i}$$

where $\beta_{0}$, $\beta_{i}$, $\beta_{ii}$, and $\beta_{ij}$ represent the coefficients of intercept, linear, quadratic, and interaction variables, respectively; $Y_{i}$ refers to the predicted response; $x_{i}$ and $x_{j}$ are defined as the independent variables in the coded unit; and $\varepsilon_{i}$ is the error term.

The comprehensive regression modeling approach is facilitated by Eq. (1), a second-order response function, which identifies the associations between the four input parameters and the three response variables. During the analysis, we identified the coefficients that resulted in the best fit for Eq. (1) through regression analysis using Minitab 20, a statistical analysis software tool. This process not only yielded a regression model explaining the statistical relationship between predictors and response variables but also retained predictors involved in higher-order terms, even if they were initially deemed insignificant.

Before proceeding with performance analysis, we employed the experimental input-output data. The design matrices guided the application of these data in constructing the models. The second-order empirical formulas for uncoded values of YS, UTS, and UE are presented in Eqs. (2), (3), (4), respectively:

(2) $$YS=308.68-1.05A-4.16B-23.45C-1.20D+7.35C^{2}-6.090AC+2.198AD$$

(3) UTS=340.26−16.34A+2.18B+3.45C+1.19D−2.90AB−1.930AC+1.680AD

(4) $$UE=38.14-10.616A-0.144C-4.99D+1.484D^{2}+0.641AC+0.610AD$$

This comprehensive regression modeling approach facilitates a thorough examination of the complex relationships between input parameters and the mechanical properties of SS400 sheet specimens, accounting for both linear and quadratic effects.

### 2.4 ANOVA for model adequacy validation

In this section, we conduct ANOVA analyses at a 95 % confidence level to investigate the impact of input parameters on the three response variables (YS, UTS, and UE) and validate the corresponding regression models. ANOVA is a statistical technique that helps us understand how variations in input factors influence the response variables and whether the developed models accurately capture these relationships.

#### 2.4.1 YS response

In Table 5, the P-values associated with terms highlighted as A, B, C, D, $C^{2}$, A × C, and A × D terms indicate a statistically significant influence on the YS response. Furthermore, the SD between the data points and the fitted values (S-values), determination coefficient (R-sq), and the adjusted determination coefficient (R^2^-adj) were examined to evaluate the accuracy of the developed model in predicting the experimental data. Based on the model summary in Table 5, the developed model demonstrated high accuracy in predicting the experimental data.

**Table 5:** ANOVA result for YS.

Source	DF	Seq SS	Contribution	Adj SS	Adj MS	F-Value	P-Value
Model	7	5577.9	94.08 %	5577.9	796.84	63.55	0.000
Linear	4	4139.7	69.82 %	4139.7	1034.92	82.54	0.000
A	1	2808.5	47.37 %	2808.5	2808.48	223.98	0.000
B	1	156.1	2.63 %	156.1	156.08	12.45	0.001
C	1	930.5	15.69 %	930.5	930.53	74.21	0.000
D	1	244.6	4.13 %	244.6	244.57	19.50	0.000
Square	1	432.2	7.29 %	432.2	432.23	34.47	0.000
*C*^*2*^	1	432.2	7.29 %	432.2	432.23	34.47	0.000
2-Way Interactions	2	1006.0	16.97 %	1006.0	503.00	40.11	0.000
A × C	1	890.0	15.01 %	890.0	890.03	70.98	0.000
A × D	1	116.0	1.96 %	116.0	115.97	9.25	0.005
Error	28	351.1	5.92 %	351.1	12.54		
Total	35	5929.0	100.00 %				
Model Summary: S = 3.54105; R-sq = 94.08 %; R-sq (adj) = 92.60 %; R-sq (pred) = 90.41 %							

**Fig. 3:**
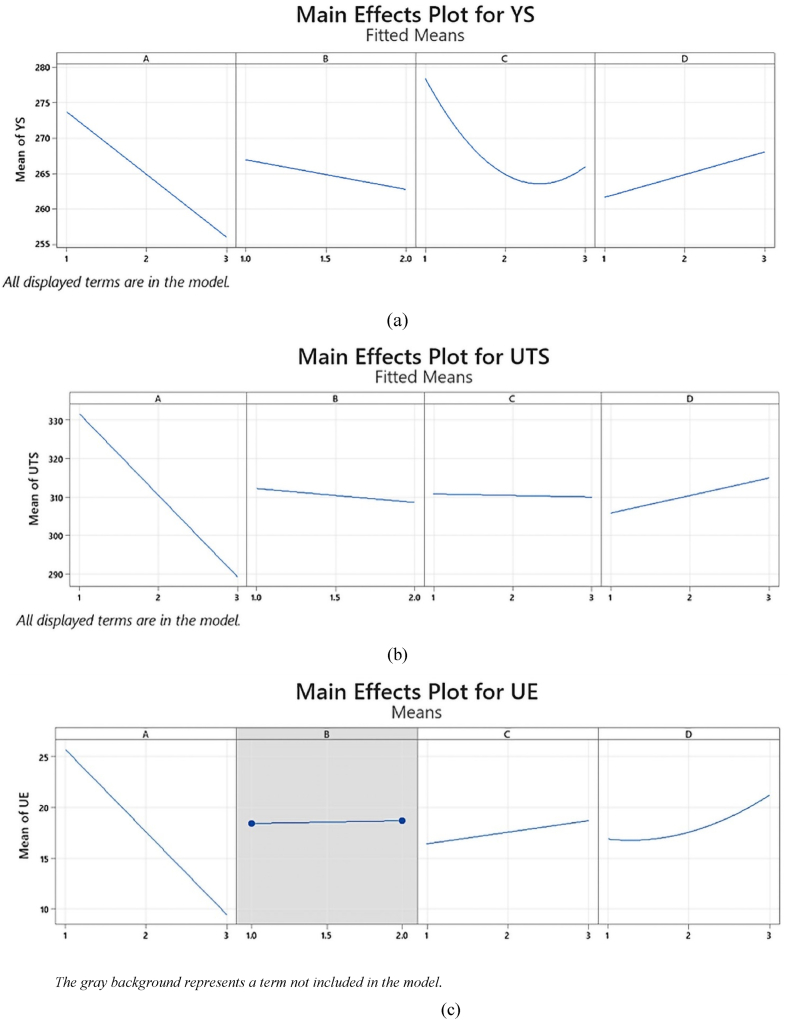
Main effects plot for (a) YS, (b) UTS and (c) UE.

Significantly, the greatest impact on YS is associated with hole diameter (47.37 %), followed by the number of holes (15.69 %), shape (4.13 %), and location (2.63 %). The square term of the number of holes ($C^{2}$) proved to be noteworthy, contributing a substantial influence of 7.29 %, with its relationship to YS identified as nonlinear (see Fig. 3a). Additionally, interaction terms demonstrated significant effects on the YS of SS400 sheet specimens. Specifically, the A × C term (diameter and number) and A × D term (diameter and shape) exhibited influences of 15.01 % and 1.96 %, respectively.

#### 2.4.2 UTS response

Table 6 presents the ANOVA result for the UTS response. Statistically significant influences on UTS are highlighted for terms A, B, D, A × B, and A × C, as indicated by their respective P-values and remarks. Conversely, term C exhibited no significant effect on the response variable (see Fig. 3b for reference). To further assess the alignment of the experimental data with the fitted regression model, an analysis of the SD between data points, S-values, R-sq, and R^2^-adj was conducted. The model summary in Table 6 indicates that the developed model exhibits high accuracy in predicting the experimental data.

**Table 6:** ANOVA result for UTS.

Source	DF	Seq SS	Contribution	Adj SS	Adj MS	F-Value	P-Value
Model	11	17237.8	97.68 %	17237.8	1567.1	91.98	0.000
Linear	6	16849.9	95.49 %	16849.9	2808.3	164.84	0.000
A	1	16163.3	91.59 %	16163.3	16163.3	948.71	0.000
B	1	117.6	0.67 %	117.6	117.6	6.90	0.015
C	2	51.0	0.29 %	51.0	25.5	1.50	0.244
D	2	518.0	2.94 %	518.0	259.0	15.20	0.000
2-Way Interactions	5	387.8	2.20 %	387.8	77.6	4.55	0.005
A × B	1	75.6	0.43 %	75.6	75.6	4.44	0.046
A × C	2	198.8	1.13 %	198.8	99.4	5.83	0.009
A × D	2	113.4	0.64 %	113.4	56.7	3.33	0.053
Error	24	408.9	2.32 %	408.9	17.0		
Total	35	17646.6	100.00 %				
Model Summary: S = 4.12760; R-sq = 97.68 %; R-sq (adj) = 96.62 %; R-sq (pred) = 94.79 %							

The analysis reveals that hole diameter exerts the highest influence (91.59 %) on UTS, followed by hole shape (2.94 %) and hole location (0.67 %). In terms of the 2-way interaction, the A × C (diameter and number), A × D (diameter and shape), and A × B (diameter and location) recorded an influence of 1.13 %, 0.64 %, and 0.43 %, respectively. Thus, the interaction terms also showed a significant effect on the UTS of the SS400 sheet specimens.

#### 2.4.3 UE response

Table 7 presents the ANOVA result in terms of the UE response. Both main and interaction effects of term B are insignificant. However, the terms A, C, D, $D^{2}$, A × C, and A × D significantly affected the response variable. The R-sq and R^2^-adj achieved in the model summary, as indicated in the model summary and representing the actual data predicted by the proposed regression model, suggest a relatively good fit. Similar to the UTS, the diameter exhibited the maximum statistical influence (91.18 %) on UE, followed by the shape (4.32 %) and number (1.20 %) of holes. The square term of the hole shape ($D^{2}$) is significant and exhibited an influence of 0.68 % (refer Fig. 3c). Table 7 also shows that the interaction between the A × C (diameter and numbers) and A × D (diameter and shape) interactions demonstrated an influence of 0.38 % and 0.34 %, respectively, on the UE of the SS400 sheet specimens.

**Table 7:** ANOVA analysis for UE.

Source	DF	Seq SS	Contribution	Adj SS	Adj MS	F-Value	P-Value
Model	7	2550.41	98.13 %	2550.41	364.34	209.84	0.000
Linear	4	2513.99	96.73 %	2513.99	628.5	361.97	0.000
A	1	2369.82	91.18 %	2369.82	2369.82	1364.85	0.000
B	1	0.69	0.03 %	0.69	0.69	0.4	0.534
C	1	31.14	1.20 %	31.14	31.14	17.93	0.000
D	1	112.35	4.32 %	112.35	112.35	64.71	0.000
Square	1	17.62	0.68 %	17.62	17.62	10.15	0.004
*D*^*2*^	1	17.62	0.68 %	17.62	17.62	10.15	0.004
2-Way Interaction	2	18.8	0.72 %	18.8	9.4	5.41	0.010
A × C	1	9.87	0.38 %	9.87	9.87	5.69	0.024
A × D	1	8.93	0.34 %	8.93	8.93	5.14	0.031
Error	28	48.62	1.87 %	48.62	1.74		
Total	35	2599.03	100.00 %				
Model Summary: S = 1.31769; R-sq = 98.13 %; R-sq (adj) = 97.66 %; R-sq (pred) = 96.86 %							

Based on the ANOVA results presented in Table 5, Table 6, Table 7, the developed regression models for YS, UTS, and UE demonstrate strong correlation coefficients of 0.9408, 0.9768, and 0.9813, respectively. The reliability and effectiveness of the proposed second-order regression models in predicting multiple responses are underscored by two critical findings: (i) all three models exhibited P-values below 0.05, signifying statistical significance, and (ii) the F-ratios for these models significantly surpassed the critical F-ratios at a 99 % confidence level. These outcomes affirm the robustness and dependability of the predictive models.

### 2.5 Verification of the prediction accuracy

In order to further substantiate the accuracy of the predicted model, we conducted ten additional experimental tests. The aim was to assess the relative deviation between the experimental output values and those predicted by the models using randomly selected input parameters from Table 4. The results of this verification process are summarized in Table 8, Table 9.

**Table 8:** Summary of the prediction test accuracy for YS.

No.	Input parameters				YS			
	A (mm)	B	C	D	Exp. (MPa)	Pred. (MPa)	Percent deviation	Abs. percent deviation
1	1	1	1	1	283	282	−0.26	0.26
2	1	1	2	2	280	276	−1.53	1.53
3	1	1	3	3	275	284	3.17	3.17
4	1	2	1	3	281	280	−0.32	0.32
5	1	2	3	1	279	278	−0.42	0.42
6	1	2	3	3	280	280	−0.06	0.06
7	3	1	1	3	281	283	0.77	0.77
8	3	1	3	2	253	253	0.06	0.06
9	3	2	1	3	276	279	1.08	1.08
10	3	2	3	3	247	254	2.90	2.90
Minimum percent deviation:							−1.53	
Maximum percent deviation:							3.17	
Average of absolute percent deviation:								1.06

**Table 9:** Summary of the prediction test accuracy for UTS and UE.

No.	Input parameters				UTS				UE			
	A (mm)	B	C	D	Exp. (MPa)	Pred. (MPa)	Percent deviation	Abs. percent deviation	Exp. (%)	Pred. (%)	Percent deviation	Abs. percent deviation
1	1	1	1	1	325	328	0.79	0.79	25.36	25.13	−0.94	0.94
2	1	1	2	2	334	332	−0.61	0.61	25.53	25.69	0.64	0.64
3	1	1	3	3	333	336	1.00	1.00	29.61	29.23	−1.30	1.30
4	1	2	1	3	332	333	0.18	0.18	28.04	28.24	0.70	0.70
5	1	2	3	1	333	330	−0.94	0.94	26.23	26.12	−0.42	0.42
6	1	2	3	3	335	336	0.19	0.19	29.02	29.23	0.72	0.72
7	3	1	1	3	298	301	1.02	1.02	11.77	11.95	1.48	1.48
8	3	1	3	2	286	290	1.43	1.43	11.19	11.25	0.49	0.49
9	3	2	1	3	287	295	2.56	2.56	11.75	11.95	1.65	1.65
10	3	2	3	3	289	290	0.30	0.30	15.48	15.51	0.16	0.16
Minimum percent deviation:							−0.94				−1.30	
Maximum percent deviation:							2.56				1.65	
Average of absolute percent deviation:								0.90				0.85

The percentage deviation for the ten predicted experimental cases ranged from −1.53 % to +3.17 % for YS, −0.94 % to +2.56 % for UTS, and −1.30 % to +1.65 % for UE, as shown in Table 8, Table 9 These ranges illustrate the variation in prediction accuracy across the different experiments. Notably, these deviations encompass both positive and negative values, indicating that the model predictions occasionally overestimated or underestimated the experimental results.

Considering the entirety of these ten experiments, we calculated the average absolute percentage deviation. The results revealed remarkably low deviations, with values of 1.06 % for YS, 0.90 % for UTS, and 0.85 % for UE (refer to Table 8, Table 9). These consistently low absolute percentage deviations underscore the practical utility of the derived equations across various industries. The robustness and accuracy of the predictive models, as indicated by these findings, reaffirm their suitability for real-world applications, where precision and reliability are paramount.

### 2.6 The CRITIC method

The CRITIC method, introduced by Diakoulaki [35], serves to evaluate the weight fractions associated with each objective function critical for addressing multi-objective optimization problems. Typically, multi-objective functions yield numerous potential solutions, each corresponding to the weights assigned to individual responses. However, allocating a maximum weight fraction to a single output may compromise the solution for other responses. The CRITIC method addresses this by integrating contrast intensity and the inherent conflicting nature within the decision-making problem to determine weight fractions. The following steps outline the process of determining objective weights using the CRITIC method:

Step 1- A decision matrix $D=\left[d_{ij}\right]$ is formulated, denoted by the output value of the alternative indexed by $i^{th}$ corresponding to the criterion indexed by $j^{th}$. Initially defined by Eq. (5), this matrix encompasses a collection of m feasible alternatives and n criteria.

(5) $$D={\left[d_{ij}\right]}_{m\times n}=\left[\begin{array}{cccc} d_{11} & d_{12} & ... & d_{11}\\ d_{21} & d_{22} & ... & d_{2n}\\ ... & ... & ... & ...\\ d_{m1} & d_{m2} & ... & d_{mn} \end{array}\right]\begin{array}{ccc} & for & i=1,2,...,m;\;\begin{array}{cc} and & j=1,2,...,n \end{array} \end{array}$$

Step 2- The formulated *D* is normalized using Eq. (6) to mitigate numerical fluctuations in the output values of various response criteria, ensuring their standardization within the range of [0,1].

(6) $$\bar{d_{ij}}=\frac{d_{ij}-d_{j}^{worst}}{d_{j}^{best}-d_{j}^{worst}}$$

where $\bar{d_{ij}}$ is the normalized output for the $i^{th}$ alternative in relation to the $j^{th}$ criterion, with $d_{j}^{worst}$ as the worst and $d_{j}^{best}$ as the best output for the same criterion.

Step 3- The contrast in criteria intensity is evaluated based on the SD of the criterion values normalized by columns (denoted as $d_{j}$), as calculated using Eq. (7).

(7) $$\sigma_{j}=\sqrt{\frac{\sum_{i=1}^{m}{\left(\bar{d_{ij}}-\bar{d_{j}}\right)}^{2}}{m}}$$

where $\bar{d_{j}}$ represents the average output value of the criterion indexed by $j^{th}$, while m signifies the total number of experiments.

Step 4- A square matrix with symmetry is constructed, incorporating the terms representing the linear correlation coefficients between the criteria $r_{jk}$, as indicated in Eq. (8).

(8) $$r_{jk}=\frac{\sum_{i=1}^{m}\left(\bar{d_{ij}}-\bar{d_{j}}\right)\left(\bar{d_{ik}}-\bar{d_{k}}\right)}{\sum_{i=1}^{m}{\left(\bar{d_{ij}}-\bar{d_{j}}\right)}^{2}\sum_{i=1}^{m}{\left(\bar{d_{ik}}-\bar{d_{k}}\right)}^{2}}$$

Step 5- The criterion information $c_{j}$ is determined from the product of Eqs. (5) and (6), as described in Eq. (9).

(9) $$c_{j}=\sigma_{j}\sum_{k=1}^{m}1-r_{jk}$$

Step 6- The normalization method is employed to determine the weights for each output based on the criterion information, as outlined in Eq. (10):

(10) $$w_{j}=\frac{c_{j}}{\sum_{j=1}^{n}c_{j}}$$

### 2.7 Desirability Function Analysis (DFA)

The mathematical model, as detailed in Eqs. (2), (3), (4), primarily comprises quadratic equations.

This model represents a small-scale problem, rendering it suitable for the precise derivation of solutions. Given the nature of the problem at hand, the DFA method emerges as an analytical approach capable of offering accurate solutions, aligning seamlessly with the central focus of this study.

DFA [52] determines the satisfactory level of the set of input parameters to the objective function, in particular, the minimization or maximization of the responses. The optimization of the composite desirability (referred to as Overall Desirability), denoted as $D_{O}$, which has a value range of [0,1], is needed for the input variable settings. Generally, a $D_{O}$ value close to 1 indicates an ideal condition for any optimization process. The $D_{O}$ value is considered a fitness function for the optimization problem and is computed using Eq. (11), considering all responses, as follows:

(11) $$D_{O}=\sqrt[{3}]{\left(d_{\text{YS}}^{W_{1}}\times d_{\text{UTS}}^{W_{2}}\times d_{\text{UE}}^{W_{3}}\right)}$$

where the terms $W_{1}$ , $W_{2}$, and $W_{3}$ refer to the CRITIC weights corresponding to the YS, UTS, and UE. In Eq. (11), each component of the composite desirability index, represented by $d_{\text{YS}}$, $d_{\text{UTS}}$, and, $d_{\text{UE}}$ reflects the individual desirability of a specific response variable. The $D_{O}$ value encapsulates the collective desirability of the entire set of input parameters. Understanding the significance of this equation is pivotal for comprehending how DFA contributes to the evaluation and optimization of the chosen parameter configurations.

Additionally, the CRITIC method was employed for determining the weights assigned to each response. Optimization of YS, UTS, and UE aimed at achieving their respective maximum values, and their desirability values were calculated using Eq. (12).

(12) $$d_{\text{YS}}=\frac{\text{YS}\;-\;{\text{YS}}_{\min}}{{\text{YS}}_{\max}-\;{\text{YS}}_{\min}},d_{\text{UTS}}=\frac{\text{UTS}\;-\;{\text{UTS}}_{\min}}{{\text{UTS}}_{\max}-\;{\text{UTS}}_{\min}},d_{\text{UE}}=\frac{\text{UE}\;-\;{\text{UE}}_{\min}}{{\text{UE}}_{\max}-\;{\text{UE}}_{\min}}$$

In these equations, the terms ${\text{YS}}_{\max}$, ${\text{UTS}}_{\max}$, and ${\text{UE}}_{\max}$ are defined as the maximum values of YS, UTS, and UE, while the terms ${\text{YS}}_{\min}$, ${\text{UTS}}_{\min}$, and ${\text{UE}}_{\min}$ represent the minimum values of YS, UTS, and UE. The terms $d_{\text{YS}}$, $d_{\text{UTS}}$, and $d_{\text{UE}}$ denote the desirability indices associated with YS, UTS, and UE, respectively.

## 3 Results and discussion

In this section, we delve into a comprehensive analysis of the results obtained from the models developed using experimental input-output data. The CRITIC method is used to establish the weight fraction for each response, while simultaneously, the DFA is employed to identify optimal parameter combinations for multiple outputs.

### 3.1 Effect of hole configurations on the tensile properties

In this subsection, we explore the impact of different hole configurations on tensile properties at different levels. To illustrate the relationships between responses and input parameters, we construct main effect plots and interaction plots.

#### 3.1.1 Main effect plots for YS, UTS, and UE

Fig. 3 displays the main effect plots designed to identify the factor levels with the most significant response values. These plots represent the mean change in all three responses (YS, UTS, and UE) as we adjust the main factor levels from low to high levels through the central point. The existence of a main effect is indicated by variations in the mean response across different levels of a factor. The reference line serves to represent the overall mean in this context.

As observed in the plotted graph, changes in YS, UTS, and UE are closely tied to hole diameter, as indicated by the steepest line. A steeper slope signifies a more substantial main effect. Notably, as the hole diameter increases, the values of YS, UTS, and UE of the specimen at failure decrease. The observed trend corresponds with Mallick's study [1], which identified a relationship between an enlarged hole diameter and a subsequent reduction in tensile strength. This decline in tensile strength is linked to the stress concentration mechanism near the holes, contributing to the initiation and propagation of cracks during the tensile test [53]. Subsequently, Brown et al. [54] also noted that an increased hole diameter to thickness ratio resulted in reduced elongation.

Hole shape also exerts a significant influence on the mean YS, UTS, and UE, as evidenced by the considerable departure from the horizontal line (though not as pronounced as the diameter's main effect). The extent of strength reduction in the material seems to be dependent on the type of specimen used [5]. In the case of the SS400 specimens in this study, the strength reduction resulting from open hexagonal, square, and circular holes in sheets subjected to tension was approximately 268, 265, and 262 MPa in mean YS, and 316, 310, and 306 MPa in mean UTS, respectively. The highest reduction in elongation was observed for the sheet with a circular hole, followed by those with the square hole and the hexagonal hole.

Furthermore, hole location exhibits slight negative changes in mean YS, from 267 to 264 MPa, and in mean UTS, from 312 to 309 MPa, as the location shifts from centric to eccentric. Hole eccentricity results in regions of failure initiation at the perforation edge in a small-side region within the hole-to-edge distance, which then expands to the large-side region. A larger hole eccentricity can lead to a greater decrease in YS and UTS, corroborating the findings of Mallick [1], which highlight the impact of large hole eccentricity on notched tensile strength. However, it's worth noting that the presence of a horizontally eccentric hole doesn't consistently decrease tensile stress, as observed in the study conducted by Wu et al. [55]. In cases where the eccentricity of the hole is positioned further away from the center point, tensile stress tends to be higher. Additionally, research by Van De Steen et al. [56] demonstrates that altering the diameter or eccentricity of a hole significantly influences both tensile stress and stress gradient at the surface of the hole. In contrast, mean UE slightly increased from 18.43 % to 18.70 % as the location shifted from centric to eccentric.

As the number of holes increased from 1 to 3, both YS and UTS experienced a decrease. Tensile stress was noted in the lateral regions of the specimen, and the extent of these stress zones varied in accordance with the number of holes. In other words, the more holes present, the wider the stress area [57]. However, an increase in the number of holes caused the smallest reduction in UE. The presence of multiple holes, from 1 to 3, fabricated in the sample parallel to the applied direction, maintaining a center-to-center distance of 6 mm, resulted in less stress concentration around the hole's circumference. This suggests that increasing the number of holes, parallel to the applied load, is effective in reducing stress concentration. It's essential to note that test results may change if the center-to-center distance falls below the specification minima.

In summary, it is evident that hole diameter significantly influences YS, UTS, and UE, while hole location, number, and shape have comparatively minor effects, as indicated by their shallower lines in the main effect plots against the x-axis. These observations align with the percentage contributions of each parameter in ANOVA, as presented in Table 5, Table 6, Table 7.

#### 3.1.2 Interaction plots for YS, UTS, and UE

Interaction plots serve as valuable tools for visualizing the effects of different factor combinations and identifying the most influential factors in our study. They also provide insights into the interactions among variables, which are essential for optimizing operating parameters in a multivariable system [58].

In these plots, we display the mean response for every possible combination of factors. The response is influenced by an interaction between two factors, where the influence of one variable varies based on the level of the other [59]. This phenomenon becomes evident on the plots as non-parallel lines [60]. Additionally, when there is an interaction effect, the combined effect may be either greater or less than the predicted value [61].

**Fig. 4:**
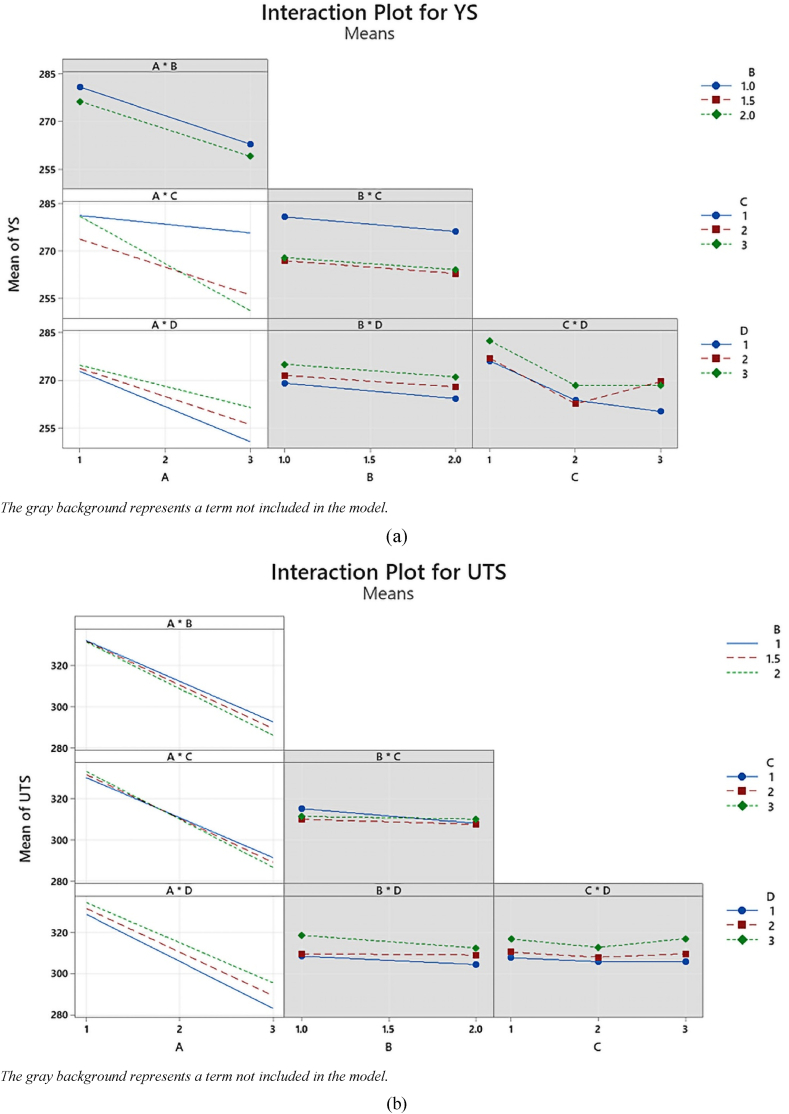

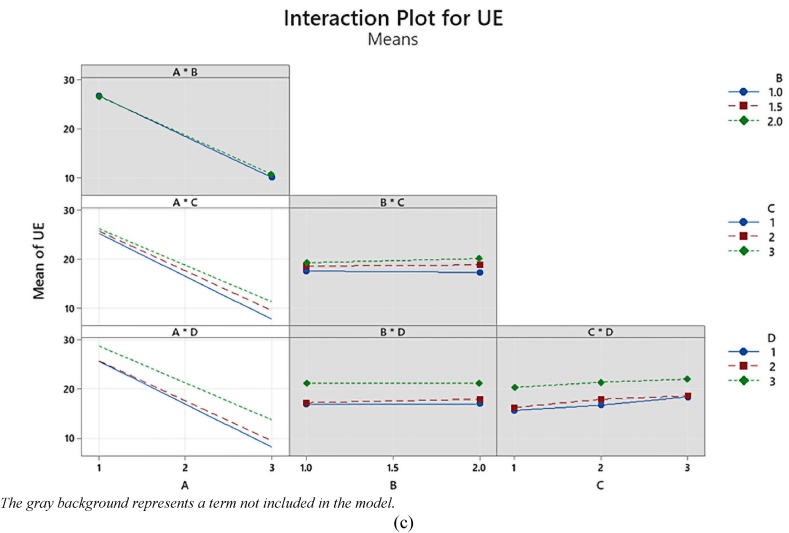
Interaction plot for (a) YS, (b) UTS, and (c) UE.

In Fig. 4, we present the full interaction plot matrix illustrating the relationships between the four factors: hole diameter (A), location (B), number (C), and shape (D) at each collocation level and their effects on YS, UTS, and UE. The matrix reveals the significant influence of hole diameter (A) on YS, UTS, and UE, indicating a robust interaction with other factors. Particularly, a strong two-way interaction is observed between hole diameter (A) and the number of holes (C) as well as hole diameter (A) and the shape of the holes (D), visually represented by non-parallel effect lines in the YS, UTS, and UE responses. In contrast, in alternate graphs across the matrix, where the lines exhibit parallelism, it suggests a feeble or insignificant interaction among the various variables regarding their impact on YS, UTS, and UE. To ascertain the statistical significance of these identified patterns, we conducted a thorough examination of the P-values linked to the interaction terms in the ANOVA tables (Table 5, Table 6, Table 7).

Fig. 4a illustrates the interaction effects on YS, focusing on two key factors, A × C and A × D, and their behavior concerning variations in A and C values. In the case of the A × C interaction, as C decreases, there is a corresponding increase in A. This relationship is particularly evident when A is set at 1, as changes in C have relatively little impact. Notably, the lines representing different levels of C diverge, signifying that as A increases, the lines decrease, although at varying rates. Conversely, the A × D interaction exhibits a different pattern. Here, as A increases, there is an increase in D, showcasing a positive correlation between the two factors. Interestingly, the red line representing the interaction A × C remains relatively stable, and for cases where A equals 1, minimal changes are observed as C increases. It's worth noting that, compared to the A × C interaction, the A × D interaction appears to have a less pronounced effect. These observations shed light on the intricate relationships between the variables and provide valuable insights into the impact of A, C, and D on YS.

Fig. 4b presents the interaction effect on UTS. In the case of interaction terms involving A × B, all three lines appear nearly parallel, except for a possible exception where the green line diverges when B = 2. Similarly, with interaction terms involving A × C, the lines remain approximately parallel, with a potential exception when C = 3. Likewise, for interaction terms involving A × D, all three lines run nearly parallel, except for a possible deviation observed when D = 3. It's worth noting that the contribution of the A × B interaction term seems to be relatively smaller compared to the contributions of A × C and A × D.

Fig. 4c showcases the interaction effect of UE, specifically focusing on the interactions involving A × C and A × D. In this plot, the variations in A, C, and D are examined. As C decreases while keeping A constant, there is an observable increase in UE, with the most pronounced effect occurring when A = 1. However, as A increases, the lines representing the three levels of C diverge, indicating that the impact of changes in C on UE varies across different A values. A similar pattern emerges when looking at interactions involving A × D, where the lines for all three levels of D are generally parallel, except for a potential exception with the green line (D = 3). Overall, the interaction patterns observed in the case of A × C are akin to those of A × D. This suggests that these interactions have similar contributions to the tensile properties under investigation.

In summary, these interaction plots provide valuable insights into how factors interact and influence the tensile properties under investigation. They help identify significant factors and interactions in our study, contributing to a deeper understanding of the relationships between variables.

### 3.2 Summary of the results from the CRITIC method

Identifying the optimal parameter combination for multiple responses, such as YS, UTS, and UE, within the framework of hole configuration poses a complex challenge, given the dynamic significance of these factors (refer to Table 5, Table 6, Table 7). For instance, while the term C was significant for UE, it proved insignificant for UTS. Similarly, interacting terms like A × B showed significance for UTS but not for UE. In real-world case studies, assigning equal or maximum weights to these outputs often results in a solution that's optimized for one aspect while potentially compromising others. To achieve an optimal solution that takes all responses into account, it's crucial to determine the weight of each response based on experimental data. For this purpose, we employed the CRITIC method, which streamlines the decision-making process without human intervention. This method considered all 36 FFD experimental trials (refer to Table 4). We normalized the output values within the range of 0 and 1 using Eq. (6), and the results of these normalized values, along with the corresponding standard deviations (SDs) for each output, are presented in Table 10 for reference (calculated using Eq. (7)).

**Table 10:** Normalized response values and their respective SDs.

Exp. no	Responses		
	YS (MPa)	UTS (MPa)	UE (%)
1	0.9338	0.3712	0.8148
2	0.8632	0.8325	0.8873
3	1.0000	0.9941	0.9992
4	0.7105	0.7675	0.8177
5	0.6386	0.8006	0.9247
6	0.7357	0.8071	1.0000
7	0.8520	0.8376	0.8886
8	0.9754	0.8978	0.9618
9	0.8803	0.9897	0.9625
10	0.8457	0.8287	0.8147
11	0.8591	0.9337	0.9256
12	0.7213	0.7618	0.9264
13	0.5915	0.7559	0.8527
14	0.5440	0.7833	0.9228
15	0.7077	0.8457	0.9617
16	0.7004	0.8134	0.8528
17	0.9459	0.9498	0.9617
18	0.8353	1.0000	0.9993
19	0.6192	0.1438	0.0744
20	0.7224	0.2117	0.0000
21	0.9082	0.5546	0.3334
22	0.3709	0.2034	0.1119
23	0.4128	0.1894	0.1114
24	0.4626	0.3226	0.4084
25	0.1098	0.1397	0.2235
26	0.2861	0.1249	0.1488
27	0.3509	0.2385	0.3710
28	0.5779	0.0909	0.0383
29	0.5986	0.1092	0.0383
30	0.8723	0.1810	0.2964
31	0.2827	0.0642	0.1489
32	0.2683	0.1537	0.1823
33	0.4334	0.2565	0.3701
34	0.0000	0.0000	0.2589
35	0.2329	0.0647	0.1858
36	0.2683	0.2770	0.5184
SD	0.2686	0.3532	0.3733

In the summary of the results from the CRITIC method, the process involves deducting one from the symmetry matrix (m×m) value obtained from Table 10. The resulting sum of all the responses is detailed in Table 11. To determine the correlation coefficient for each criterion, we applied Eq. (8), and then, the weights for individual outputs were calculated using Eqs. (9), (10). The outcomes of this analysis revealed that the weights corresponding to YS, UTS, and UE were found to be equal to 0.3825, 0.2559, and 0.3616, respectively. This information summarizes key findings from the CRITIC method, offering insights into the criteria's relative importance in the study's context.

**Table 11:** The correlation coefficient, criterion information, and weights of individual outputs.

	Correlation coefficient			Criterion information $c_{j}$	Weights $w_{j}$
	YS	UTS	UE		
YS	1.0000	0.7456	0.6404	0.1649	0.3825
UTS	0.7456	1.0000	0.9419	0.1103	0.2559
UE	0.6404	0.9419	1.0000	0.1559	0.3616

### 3.3 Summary of multi-objective optimization via DFA

In this summary of multi-objective optimization via DFA, our study's primary objective was to assess the potential of input parameters to maximize YS, UTS, and UE for open holes in the SS400 sheet specimen. We derived a response equation that is statistically reliable and robust enough to serve as the objective function for determining the optimal parameter set. Consequently, we employed DFA to identify the optimal input parameters that lead to the maximization of these multiple responses. Utilizing the regression equation, as described in Eqs. (13), (14), (15), (16), according to the input variable constraints:

(13) 1≤diameter≤3

(14) 1≤location≤2

(15) 1≤number≤3

(16) 1≤shape≤3

When considering the constraints on input variables, we determined the input parameters with the greatest potential for enhancing YS, UTS, and UE and achieving the highest levels of these tensile properties in SS400 sheet specimens. These findings support the notion that our approach is not only statistically sound but also practically applicable for optimizing multi-objective scenarios.

This section summarizes the crucial steps and findings regarding the multi-objective optimization process conducted using DFA, providing insights into the optimal parameter values to enhance tensile properties.

Using Minitab 20 software, we implemented an optimization approach based on DFA, utilizing the process parameters and responses studied in this research. The software provides a comprehensive overview of the optimization goals by presenting the design parameters for each response in Table 12. These results were carefully scrutinized to ensure the accuracy of the displayed design parameters. Equal importance was assigned to all responses, represented by a value of 1, for the sake of simplicity and impartiality. The goals, lower limits, targets, and weights were defined for each individual response, forming the basis of the desirability function. Table 11 provides an overview of the weights assigned to each response, which were subsequently utilized in the calculation. In this study, our primary objective for all responses was to maximize their values. Consequently, we set targets for optimization with the aim of achieving the highest values for these responses. For YS, we set a benchmark of excellence at 288.449. Values falling below 240.000 were considered unacceptable in this context. Similarly, in the case of UTS, we established an excellent value of 340.540, while values below 277.610 were classified as unacceptable. Regarding UE, with an excellent value of 28.637, and values below 5.554 were considered unacceptable. These well-defined parameters and targets provided the essential framework for our optimization process, ensuring our focus was on achieving the highest possible values for these critical responses.

**Table 12:** Optimization goals.

Response	Goal	Lower	Target	Weight	Importance
YS	Maximum	240.000	288.449	0.3825	1
UTS	Maximum	277.610	340.540	0.2559	1
UE	Maximum	5.554	28.637	0.3616	1

**Fig. 5:**
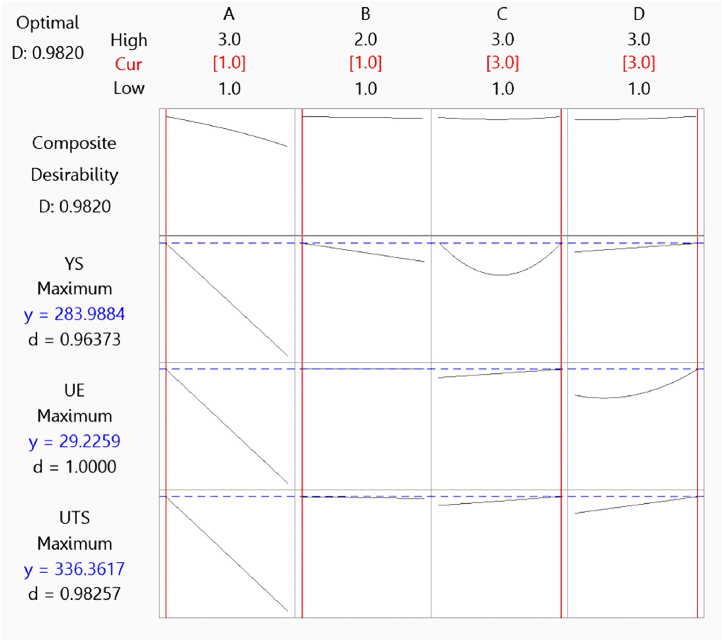
Multi-response optimization plot for maximum tensile properties.

The multi-response optimization plot in Fig. 5 was generated using Minitab software. Optimization of process parameters for YS, UTS, and UE is achieved through composite desirability. The overall composite desirability score is 0.9820, with individual desirability values of 0.96373 for YS, 0.98257 for UTS, and 1.0000 for UE. These settings correspond to the responses achieved at the maximum points of YS, UTS, and UE, with targets set at 284 MPa, 336 MPa, and 29.23 %, respectively. These target values were calculated as follows: A = 1.0, B = 1.0, C = 3.0, and D = 3.0, representing a 1 mm hole diameter, centric hole location, three holes, and a hexagonal shape.

As shown in Fig. 5, both hole diameter and shape significantly impact all responses. An increase in hole diameter from 1 mm to 3 mm generally results in a decrease in all responses. The hole's location also influences tensile properties, with eccentric locations leading to lower UTS and YS compared to centric ones. UE, however, doesn't exhibit a consistent trend with hole location. The impact on YS varies across different configurations, while the shape of the hole plays a role in the tensile properties. In most cases, hexagon-shaped holes tend to provide higher UTS and UE compared to circles and squares. UE shows some variations, and the impact of hole shape on UE depends on other factors as well. The number of holes in a specimen affects tensile properties, generally resulting in higher UTS and UE with an increase in the number of holes. However, YS doesn't exhibit a clear trend with the number of holes; it varies across different configurations.

The presented results offer valuable insights into multi-response prediction concerning variable settings, including the number of holes, hole diameter, location, and shape, and their influence on YS, UTS, and UE. These findings are pivotal for unraveling the intricate relationships between input parameters and response variables, facilitating the optimization process and informed decision-making.

In statistical analysis, as shown in Table 13, various measures assess the quality of models and predictions [62]. The SE fit, or standard error of the fit, measures the variability in the estimated mean response for specific variable settings, offering insight into the precision of predictions. A smaller SE fit indicates a more precise predicted mean response. The confidence interval (CI) for the mean response, derived from SE fit, offers a likely range of values for the mean response within a set of predictor settings. A 95 % confidence level assures us that this interval contains the population mean, aiding in evaluating the practical significance of results.

**Table 13:** Multi-response prediction for maximum tensile properties.

Response	Fit	SE Fit	95 % CI	95 % PI
YS	283.99	1.82	(280.26, 287.71)	(275.83, 292.14)
UTS	336.36	2.50	(331.23, 341.49)	(325.36, 347.36)
UE	29.226	0.634	(27.930, 30.522)	(26.261, 32.191)

Unlike the confidence interval (CI), which assesses the precision of the mean response, the prediction interval (PI) evaluates the accuracy of individual predictions for distinct variable combinations. The PI tends to be broader than the CI, reflecting the heightened uncertainty associated with predicting individual responses as opposed to the mean response. A 95 % PI offers confidence that a single response will fall within the interval given the specified predictor settings. The composite desirability, measured on a scale from 0 to 1 (where 1 denotes an ideal result and 0 signifies that one or more responses exceed acceptable boundaries), plays a crucial role in optimizing multiple responses simultaneously, evaluating how well settings align with all responses.

• For YS, the fit value of approximately 284 MPa under specified parameter settings suggests a high degree of confidence in this prediction, with a narrow 95 % CI (280.26 MPa–287.71 MPa) indicating a precise estimate. The SE fit for YS is 1.82, reflecting relatively low uncertainty associated with the model's fit. However, the broader 95 % PI (275.83 MPa–292.14 MPa) accounts for potential variability in future observations, emphasizing the importance of acknowledging this uncertainty in decision-making.
• For UTS, the fit value of around 336.36 MPa with specified parameters comes with a narrow 95 % CI (331.23 MPa–341.49 MPa), signifying a high likelihood that the actual UTS falls within this range. The SE fit for UTS is 2.50, indicating the level of uncertainty associated with the model's fit. However, the wider 95 % PI (325.36 MPa–347.36 MPa) acknowledges the potential variability in future UTS measurements, underscoring the importance of considering uncertainty.
• Regarding UE, the fit value of approximately 29.23 % under the same parameter conditions is accompanied by a narrow 95 % CI (27.930 %–30.522 %), indicating a high level of confidence in this prediction. The SE fit for UE is 0.634, indicating relatively low uncertainty in the model's fit. The wider 95 % PI (26.261 %–32.191 %) accounts for potential variability in future UE measurements, highlighting the need to consider uncertainty.

These results offer predictions for the mechanical properties of the SS400 sheet specimen based on the model, with fit values indicating a close approximation to observed data and 95 % confidence intervals offering a likely range for true values. The inclusion of SE fit values provides additional insights into the precision of the model's fit. While the chosen settings and methodology have led to highly desirable outcomes, it's crucial to consider potential sensitivity to parameter variations and the importance of precise goal-setting and weighting determination.

### 3.4 Confirmation experiment

The DFA-recommended optimal parameter conditions, including a hole diameter of 1 mm, a centric hole location, three holes, and a hexagonal hole shape, underwent experimental assessment to validate the optimization method's correspondence to the output performance. The experimental output data yielded values of 275 MPa, 333 MPa, and 29.61 % for YS, UTS, and UE, respectively.

Furthermore, a comparison between the predicted and experimental values revealed a percent error of 3.17 % for YS, 1.00 % for UTS, and 1.30 % for UE. These results validate the adequacy of the optimization approach utilizing DFA. The optimal parameter conditions identified through this method can be reliably adopted to enhance the tensile properties of open holes in SS400 sheet specimens.

## 4 Conclusion

The study has successfully demonstrated the optimization of input parameters to assess the influence of open holes on the tensile properties of SS400 sheet specimens, primarily through the integration of FFD-CRITIC-DFA method.

The FFD-based experiment yielded valuable insights, allowing the construction of experiment-based prediction models. The statistical evaluation through ANOVA revealed that all the main effect factors, namely hole diameter, location, number, and shape, were statistically significant for all responses, except the number for UTS and the location for UE. Notably, the diameter contributed the most to YS, while the number of holes followed closely. In terms of UTS and UE, the diameter had the highest influence, with the hole shape also contributing significantly. The quadratic models for all responses exhibited a high degree of fitting, with correlation coefficient values approaching 1. Moreover, the models demonstrated remarkable accuracy, as evidenced by the average absolute percent deviations of 1.06 % for YS, 0.90 % for UTS, and 0.85 % for UE across the ten experimental cases.

Furthermore, the main effects plot underscored the pivotal role of hole diameter in shaping the tensile performance of specimens with open holes. Smaller hole diameters led to higher YS, UTS, and UE values. Among these parameters, a specimen featuring three hexagonal holes, each eccentrically located with a 1 mm diameter, resulted in the least reduction in UTS and UE. In contrast, a specimen featuring a centrally located hexagonal hole with a 1 mm diameter showed the least reduction in YS.

The CRITIC method was employed to estimate the weight fractions for YS, UTS, and UE from experimental data, yielding values of 0.3825, 0.2559, and 0.3616, respectively. Subsequently, the DFA-derived composite desirability, measuring at 0.9820, provided a comprehensive measure of desirability for the recommended optimal parameter conditions. These conditions include a 1 mm hole diameter, centric hole location, three holes, and a hexagonal shape, with the objective of achieving the maximum tensile properties. The experimental validation of these optimal parameters closely matched the predicted values, demonstrating a percent error of 3.17 % for YS, 1.00 % for UTS, and 1.30 % for UE. These results affirm the adequacy of the optimization approach employing DFA. Consequently, the optimal parameter conditions identified through this integrated approach can be confidently adopted to enhance the tensile properties of open holes in SS400 sheet specimens.

In summary, this study, driven by an integrated FFD-CRITIC-DFA method, offers valuable insights into optimizing open hole configurations, with results that support its potential for enhancing the tensile performance of materials. These observations underscore the significance of considering hole configurations in engineering and design, as they have a substantial impact on the mechanical properties of SS400 sheet specimens. Further exploration and analysis of the intricate relationships and trade-offs between these factors will enable more informed decision-making in practical applications.

### Data availability

The dataset is incorporated within the article, supplementary materials, and is referenced throughout the manuscript.

## CRediT authorship contribution statement

**Teerapun Saeheaw:** Writing – original draft, Writing – review & editing, Visualization, Validation, Supervision, Software, Resources, Project administration, Methodology, Investigation, Funding acquisition, Formal analysis, Data curation, Conceptualization.

## Declaration of competing interest

The authors declare that they have no known competing financial interests or personal relationships that could have appeared to influence the work reported in this paper.
